# A Novel Fabricating Process of Catalytic Gas Sensor Based on Droplet Generating Technology

**DOI:** 10.3390/mi10010071

**Published:** 2019-01-20

**Authors:** Liqun Wu, Ting Zhang, Hongcheng Wang, Chengxin Tang, Linan Zhang

**Affiliations:** 1 School of Mechanical Engineering, Hangzhou Dianzi University, Hangzhou 310018, China; wuliqun@hdu.edu.cn (L.W.); 172010042@hdu.edu.cn (T.Z.); zhanglinan@hdu.edu.cn (L.Z.); 2 School of Media and Design, Hangzhou Dianzi University, Hangzhou 310018, China; tcx@hdu.edu.cn

**Keywords:** gas sensor, micropellistor, microdroplet, pulse inertia force, methane

## Abstract

Catalytic gas sensors are widely used for measuring concentrations of combustible gases to prevent explosive accidents in industrial and domestic environments. The typical structure of the sensitive element of the sensor consists of carrier and catalyst materials, which are in and around a platinum coil. However, the size of the platinum coil is micron-grade and typically has a cylindrical shape. It is extremely difficult to control the amount of carrier and catalyst materials and to fulfill the inner cavity of the coil, which adds to the irreproducibility and uncertainty of the sensor performance. To solve this problem, this paper presents a new method which uses a drop-on-demand droplet generator to add the carrier and catalytic materials into the platinum coil and fabricate the micropellistor. The materials in this article include finely dispersed Al_2_O_3_ suspension and platinum palladium (Pd-Pt) catalyst. The size of the micropellistor with carrier material can be controlled by the number of the suspension droplets, while the amount of Pd-Pt catalyst can be controlled by the number of catalyst droplets. A bridge circuit is used to obtain the output signal of the gas sensors. The original signals of the micropellistor at 140 mV and 80 mV remain after aging treatment. The sensitivity and power consumption of the pellistor are 32 mV/% CH_4_ and 120 mW, respectively.

## 1. Introduction

**Figure 1:**
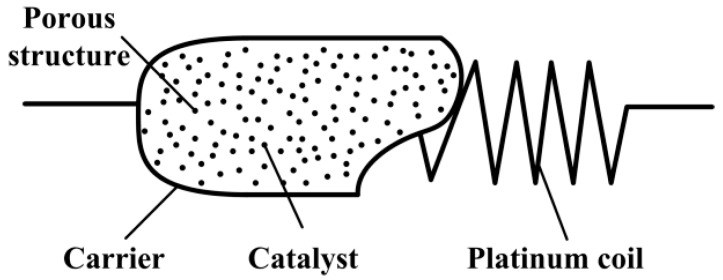
Structure of the micropellistor in a catalytic gas sensor.

Catalytic combustion type gas detectors that operate on catalytic oxidation of combustible gases are widely used for detecting gas concentration and maintaining it at below the lower explosion limit (LEL) [1]. It is an effective means to prevent explosive accidents in industrial and domestic environments [2,3]. The combustive gases include hydrogen (H_2_), methane (CH_4_), carbon monoxide (CO), organic vapors, etc. As is shown in Figure 1, the widely applicable structure of the sensitive element (called a pellistor) in the catalytic combustion type gas detectors consists of a porous structure and a platinum coil [4,5]. Therefore, the pellistor is a kind of solid phase gas sensor [6] and has a much higher sensitivity, though much effort has been put into developing a silicon microheater potentially with a high-temperature and low-power consumption [7,8]. The porous structure, constructed around the platinum coil, is called a carrier. On the inner surface is catalyst which has a catalytic effect during the detecting process. The platinum coil can heat the catalyst to a sufficiently high temperature, at which any flammable gas molecules present can produce flameless combustion [9,10] and release combustion heat. Besides that, the Pt coil serves not only as a catalyst heater, but also as a resistance thermometer.

The surface of the carrier is a porous structure, the precursor of which is nano-scale particle suspension (typically nano-scale Al_2_O_3_ suspension [11,12]) and can form the porous structure after evaporation of the solvent. Wang et al. [5] researched performances of sensors with different carriers, including Al_2_O_3_, n-Al_2_O_3_, and n-Ce-Al_2_O_3_. The carrier and catalytic materials were coated onto the platinum coil via the sometric impregnation method. 

The catalytic material is immersed into the inner-surface of the porous structure to form a large amount of catalyst activated points, which determine the sensitivity of the sensor. The greater the number of activated points the catalyst is on, the higher the sensitivity of the sensor [13].

However, the size of the platinum coil is micron-grade and typically cylindrically shaped. Therefore, coating the platinum coil with carrier and catalytic materials appeared to be an extremely critical step in practice. The cumbersome dip and drop technique [14], a thick film processing step, was adopted to coat the catalytic materials. Unfortunately, it was very difficult to control in the case of viscous species and to fulfill the inner cavity of the coil, which adds to the irreproducibility and uncertainty of the fabrication process and causes unstable performance. To solve this problem, this paper proposes a new method which uses a drop-on-demand droplet generator to introduce the carrier and catalytic materials into the platinum coil and fabricate the micropellistor.

## 2. Materials and Methods 

### 2.1. Materials

**Figure 2:**
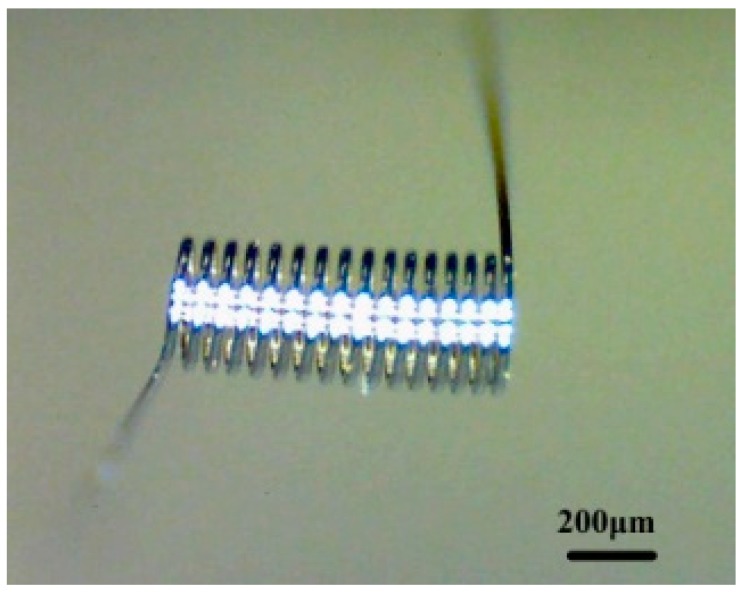
Structure of platinum coil.

The platinum coil was wound with coil diameter of 250 μm and a length of 800 μm, as shown in Figure 2. The diameter of the platinum wire was about 25 μm. The catalytic materials used included finely dispersed Al_2_O_3_ suspension and platinum palladium (Pd-Pt) catalyst.

The catalytic materials used included finely dispersed Al_2_O_3_ suspension and platinum palladium catalyst, which were ejected into the platinum coil successively. The catalytic materials needed to exhibit appropriate viscosity and stability, so the catalytic materials—especially the Al_2_O_3_ suspension—required homogeneous mixing before being used, and the optimum viscosity of the catalytic materials was below 100 cp.

### 2.2. Methods

#### 2.1.1. Droplet Generating Method Based on Pulse Inertia Force

One of the main tasks of this work is to propose a droplet generator to eject catalytic materials into the platinum coil. Droplet generating technologies mainly include two classes: continuous mode and drop-on-demand (DOD) mode. In recent years, the DOD mode has gradually replaced the continuous mode because of its promising better manufacturing control and better control of ejection time, position, and volume by a function generator. The DOD mode droplet generators include piezoelectrically [15], thermally, pneumatically, electrostatically, pulse electromagnetic force [16], and membrane-piston [17] actuated generators. The piezoelectric droplet generator has an ink containing chamber with piezoelectric elements on one or two chamber external surfaces. Displacement of piezoelectric elements can change the volume of the chamber, generate pressure waves, and eject a droplet from an orifice while the elements are applied with a pulse voltage signal. However, it is difficult for the above DOD droplet generators to be applied in ejecting the catalytic materials in this article, because the catalytic materials may corrode the nozzle, and most of the nozzles are made of metallic materials.

**Figure 3:**
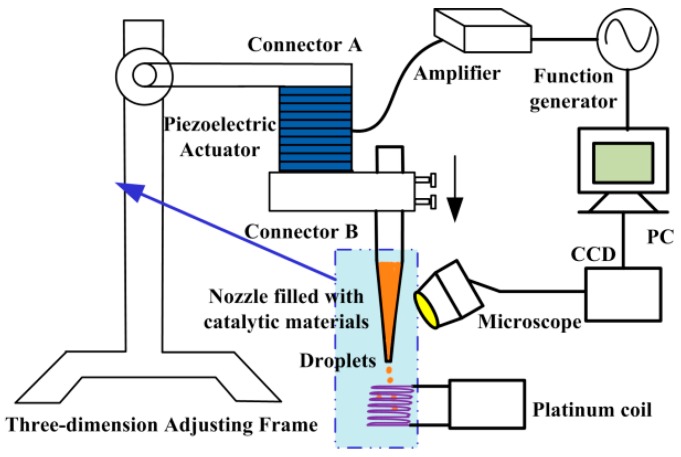
Schematic of the droplet generating device for coating catalytic materials.

As is shown in Figure 3, an apparatus for producing catalytic material droplets in an air environment was fashioned. The nozzle filled with catalytic materials was clamped by connector B, which was fixed to the bottom face of a lead zirconate titanate (PZT) stack actuator (PAL200VS25, NanoMotions, Shanghai, China) through connector A, while the upper face was fixed to a three-dimensional adjustable frame and kept stationary through the connector. There was an approximate linearity between applied voltage amplitude and the displacement of the bottom face of the actuator. Therefore, the actuator instantaneously caused a larger displacement and consequently provided a greater pulse inertia force [18] for the nozzle and catalytic materials inside when applying a higher pulse driving voltage. When the pulse inertia force was large enough and exceeded the viscous force, a droplet of liquid was ejected from the micro-nozzle drop by drop in the direction of inertial force. Pulse inertia force had no influence on the catalytic material, while the piezoelectric micro-dispenser pushed/squeezed the catalytic material, and the thermal micro-dispenser heated material to above 300 °C to form micro-droplets. Both micro-dispensers may have changed the properties of catalytic material.

**Figure 4:**
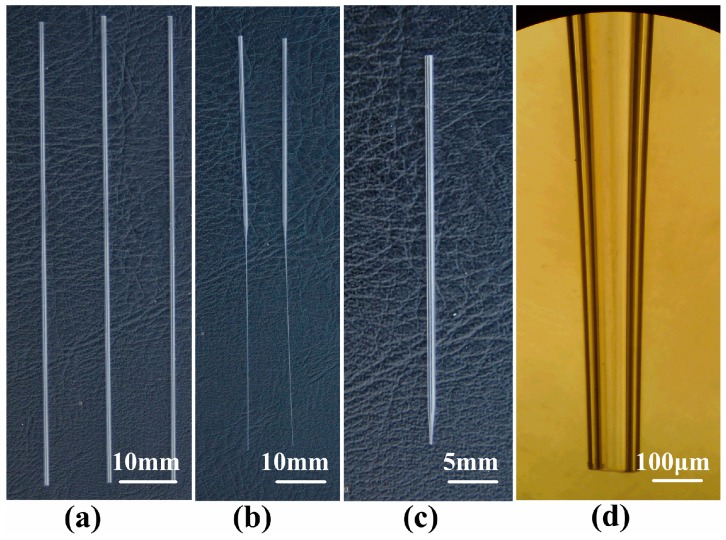
Fabrication of the borosilicate glass micro-nozzle: (**a**) borosilicate glass pipe; (**b**) micro-nozzle after being pulled; (**c**) micro-nozzle after being cut; (**d**) micrograph of micro-nozzle after being cut.

Glass material was chosen to make the tapered glass capillary because of several advantages, such as good chemical resistance, smooth surface, ease of manufacture and observation, and low cost. The raw material was borosilicate glass capillary (Beijing Zhengtianyi Scientific and Trading Co., Ltd., Beijing, China). The dimensions of the glass capillary were 1.0 mm, 0.6 mm, and 100 mm in external diameter, internal diameter, and length, respectively, as is shown in Figure 4a. A glass heating process was adopted to fabricate the micro-nozzle without complicated micro-fabrication technology and can be divided into two steps: (1) pulling a capillary to form a micro-nozzle with a straight outlet, as is shown in Figure 4b, and (2) forging the straight outlet to form a shrinkage one, as is shown in Figure 4c. The micro-nozzles with different outlet diameters were obtained by varying the control parameters (the outlet diameter in this article means the inner diameter of the nozzle tip). The fabricated micro-nozzle with an outlet diameter of 100 μm is shown in Figure 4d. The raw material of the micro-nozzle was borosilicate glass which, having good chemical inertness, allowed for no chemical reactions to occur between the catalytic materials and nozzle; conversely, most of the nozzles of micro-dispensers on the market are made of metal material.

#### 2.2.2. Manufacturing Process of the Pellistor

**Figure 5:**
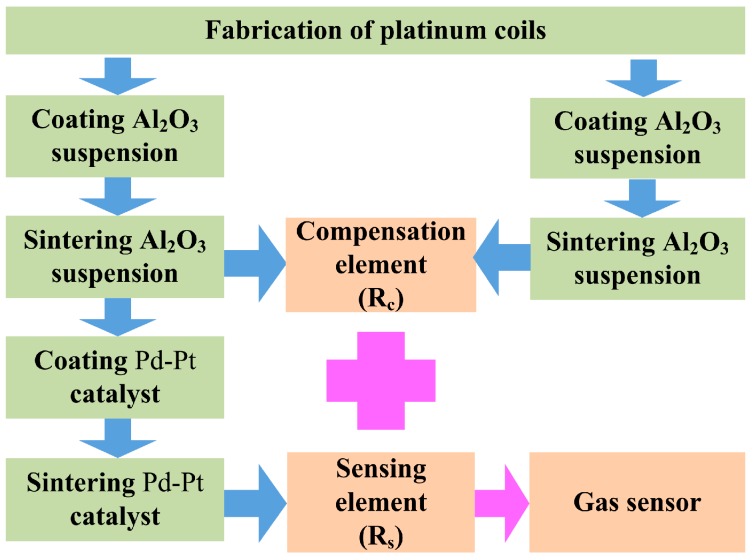
Manufacturing process of the catalytic gas sensor.

As is shown in Figure 5, the fabrication process of the pellistor using the droplet generator proposed above included the following:

(1) Adding Al_2_O_3_ suspension

**Figure 6:**
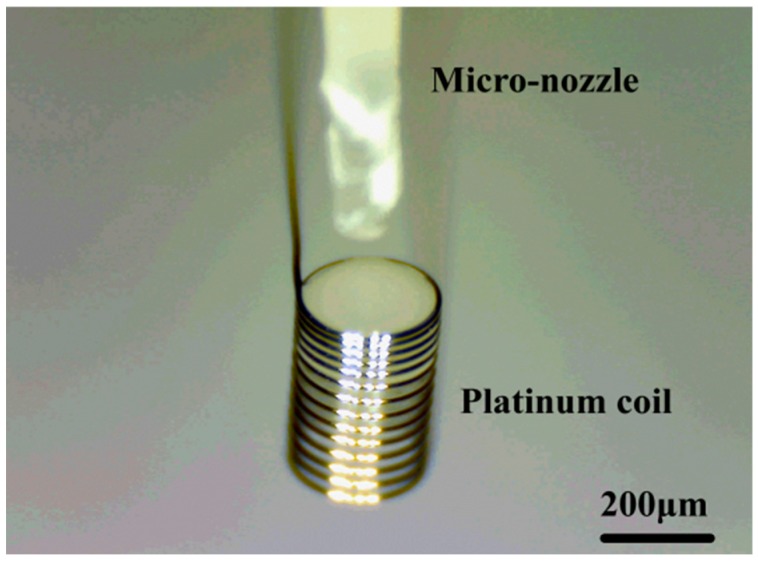
Relative position between the platinum coil and micro-nozzle.

As is shown in Figure 6, the platinum coil cylinder axis and micro-nozzle were kept vertical. The distance between the coil and the micro-nozzle was less than 2 mm to avoid forming satellites. The micro-nozzle was made to inhale a certain amount of Al_2_O_3_ suspension by a negative-pressure apparatus. The PZT function generator and amplifier was then started to excite enough inertia force for the Al_2_O_3_ suspension which was ejected droplet by droplet from of the nozzle orifice. The driving voltage and frequency were in the range of 0–80 V and 1–256 Hz, respectively. The droplet size was controlled by the driving voltage signal and orifice diameter of the micro-nozzle. The ejected Al_2_O_3_ suspension covered the entirety of the platinum coil and avoided creating a hole defect inside of the carrier.

(2) Formation of the carrier

The added Al_2_O_3_ suspension was sintered to form a porous Al_2_O_3_ matrix (γ-Al_2_O_3_ layer) by self-heating of the underlying platinum coil. The porous Al_2_O_3_ matrix was called the carrier. The resulting alumina structure established a perfect thermo-mechanical contact to the platinum coil in order to form an outer surface with sufficient temperature for catalysis and to conduct heat, which was developed by the catalytic combustion of the present gas, to the coil resistor, acting as a temperature sensor.

The sintering temperature was set at 750 °C in the experiment and the temperature holding time was twenty minutes. The temperature was controlled by the parameter of the voltage applied to the platinum coil and can be calculated by Equation (1):

(1) $$\frac{R-R_{0}}{T-T_{0}}=R_{0}\times k$$

where *k* is the temperature coefficient of resistance of platinum, a constant of 0.0026/°C. *T*_0_ is the room temperature, and *R*_0_ is the resistance of platinum at room temperature. The platinum coil with porous Al_2_O_3_ matrix can be used as a compensation element.

(3) Adding catalytic material

The micro-nozzle was made to draw in a certain amount of catalytic material by a negative-pressure apparatus, just like in step (1). The ejected catalyst soaked into and adhered to the porous structure as soon as it made contact with the matrix. The amount of added catalytic material was controlled by the size and number of the liquid droplets. 

(4) Formation of the pellistor

The matrix with catalytic material was sintered again at 550 °C to form the Pt-Pb/Al_2_O_3_ layer, the platinum element being the pellistor and the sensing element (*R_s_*). 

## 3. Results and Discussion

### 3.1. Pattern of the Pellistor

**Figure 7:**
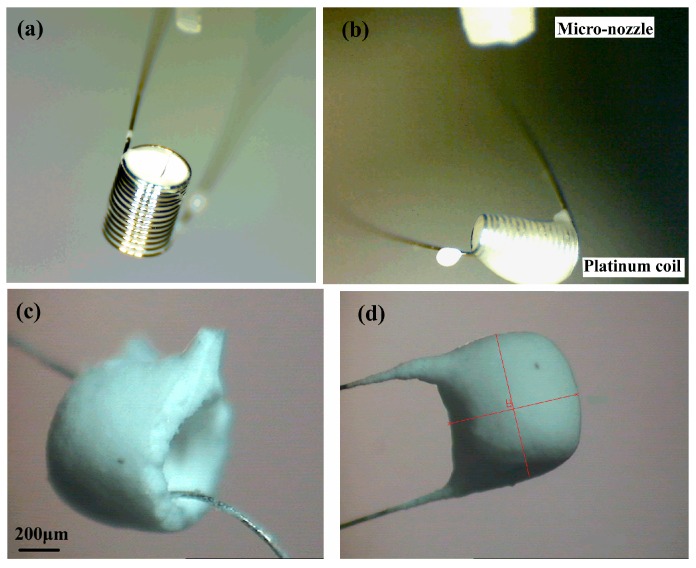
Pellistors: (**a**) filled with suspension, (**b**) with suspension on external surface, (**c**) with defects of shrinkage cavity, and (**d**) with perfect Carrier.

**Figure 8:**
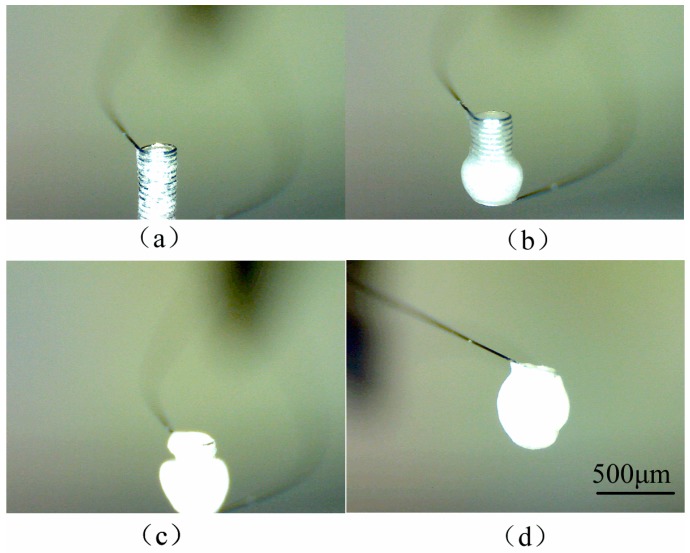
Process of coating Al_2_O_3_ suspension onto the platinum coil.

The solvents including deionized water and alcohol volatilized during the additive process. It was necessary to control the droplet generating frequency and let solvents of ejected droplets have enough time to volatilize. While adding the Al_2_O_3_ suspension, the platinum coil was first placed with its axis in the same direction of the nozzle axis. After the internal space of the coil was filled with suspension (Figure 7a), the platinum coil was placed with its axis perpendicular to the nozzle axis (Figure 7b). If the Al_2_O_3_ suspension additive speed is too high for the solvents to volatilize, a hole defect may occur, as is shown in Figure 7c. Both the inside and outside of the platinum coil was coated with the Al_2_O_3_ matrix layer. If there is no matrix outside of the coil, the platinum exposed in an air environment will oxide rapidly since it is a micro-heater itself. Therefore, after the coil was filled with Al_2_O_3_ matrix, the suspension was ejected onto the lateral surface of the columniform coil. The platinum coil with a complete Al_2_O_3_ matrix layer is shown in Figure 7d. When the concentration of the suspension was relatively low, this lower viscosity suspension flowed out of the coil, as is shown in Figure 8. In this condition, it was not necessary to change the coil axis direction during the additive process. Satellite droplets [19] were inevitable during the droplet generating process, but did not affect the pattern of the pellistor.

### 3.2. Performance of the Pellistor

#### 3.2.1. Flameless Catalytic Combustion

Methane (CH_4_), one of the most difficult-oxidative hydrocarbon combustible gases, was chosen as the testing object for the pellistor. The platinum coil heated the catalyst up to a sufficiently high temperature, at which methane gas molecules present produced flameless combustion and released combustion heat. The chemical equation for the flameless catalytic combustion is as follows, with the platinum palladium (Pd-Pt) solution as the catalyst:

(2) $${\mathrm{CH}}_{4}{\;+\;2O}_{2}\;\overset{\mathrm{Pt},\;\mathrm{Pd}}{\underset{\Delta }{\to }}{\mathrm{CO}}_{2}\;+\;{2H}_{2}O\;+\;795.5\;\mathrm{KJ}$$

The reaction product was carbon dioxide (CO_2_) and H_2_O. The higher the concentration of methane gas molecules, the more heat was released. The resistance value of platinum coil increased with the raising of the ambient temperature. Therefore, a definite numerical relationship exists between methane concentration and platinum wire resistance value.

#### 3.2.2. Activation of the Pellistor

**Figure 9:**
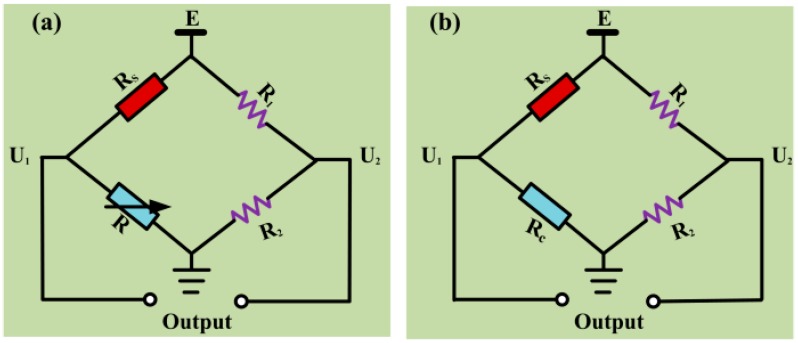
Direct current bridges: (**a**) for original signal testing, and (**b**) for steady signal testing with temperature compensation.

When methane concentration was relatively high, typically above 80% LEL (low explosion limit), the PdCl_2_ on the γ-Al_2_O_3_ layer decomposed to Pb and PbO, both of which having very high activities. An LEL of 80% LEL means that the volume fraction of CH_4_ is 4%, as the LEL for CH_4_ is 5%. It is a common method to enhance the output signal of sensor, the process of which is called sensor activation. The output signal of the pellistor was tested by a direct current bridge, as is shown in Figure 9a. The direct current bridge was composed of a pellistor (called the sensing element, *R_s_*), an adjustable resistor (*R*), and two fixed resistors (*R*_1_ and *R*_2_) at room temperature. *E*, the applied voltage of the bridge, was set at 2.6 V. The output signal (Δ*U* = *U*_1_ – *U*_2_) was set to zero by adjusting the resistance of *R* to that of *R_s_*. When sample gas flowed to the sensor, gas molecules adsorbed onto the sensitive element, combusted, and induced a temperature increase in the presence of platinum palladium catalyst. The temperature increase was short-lived compared to the increase in resistance and the output signal (Δ*U*). Moreover, this circuit did not have any amplifiers, filters, or signal process circuits. Therefore, the output was the original signal.

Therefore, the output signal Δ*U* can be calculated by Equation (3):

(3) $$\Delta U=\frac{R_{s}}{R_{s}+R}E-\frac{R_{2}}{R_{1}+R_{2}}E$$

**Figure 10:**
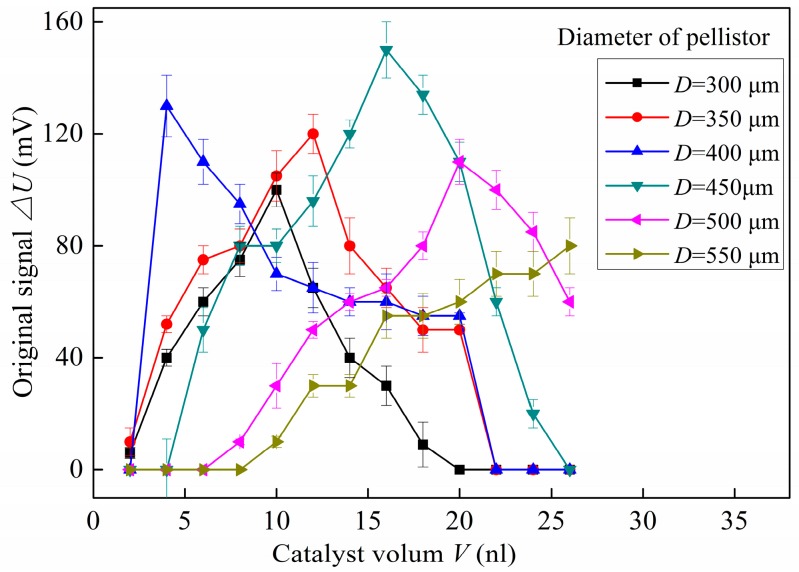
Variations in the original signals of the pellistor with porous matrix based on pellistor size and the amount of platinum palladium (Pd-Pt) catalyst.

Figure 10 shows the variation of original signal of the pellistor with porous matrix by size and the amount of Pd-Pt catalyst (sample number is 10). The porous matrix size was characterized by the diameter of the pellistor (*D*, μm), and the amount of Pd-Pt catalyst was characterized by its volume (*V*, nL). If the diameter of the pellistor was too small (*D* = 300 μm), the number of catalyst activated points was not enough for the pellistor to work a long time, and the original signal was also relatively low (below 100 mV); if the diameter of the pellistor was too large (*D* = 500 μm or 550 μm), the matrix and catalyst did not sinter completely and had poor heat transfer, which reduced the output voltage value of the sensor (below 110 mV). If the amount of the catalyst was too large, the matrix surface could not accommodate much catalyst, and consequently, the redundant catalyst caused a short-circuit while voltage was applied to the pellistor; if is the amount was too small, this small amount of catalyst (less than 7.0 nL) could not soak into whole matrix and remained only on surface. The temperature of the matrix surface layer was far below 750 °C while being sintered due to its poor heat transfer and could not form effective Pd-Pt/γ- Al_2_O_3_ layer. The maximum value of original signal was above 140 mV when the pellistor diameter and catalyst volume were 450 μm and 16.0 nL, respectively. The relative deviations of all testing data were below 5%, which means that the original signal of the pellistors had high reproducibility and stability.

#### 3.2.3. Sensitivity of the Pellistor

Aging treatment was a key process to test the performance of sensor. In aging treatment experiments, the fabricated pellistor was placed in a testing chamber flushed by standard CH_4_ gas with 0% LEL, 20% LEL, 35% LEL, 50% LEL, 75% LEL, and 100% LEL, in turn. CH_4_ gas with 0% LEL was flushed through the chamber for 2 min, and then 35% LEL was flushed through until the output signal reached 90% the standard signal (original signal). The duration of this process was called the response time. Every pellistor was tested in different CH_4_ concentrations. Each pellistor had its own chamber, and several pellistors were treated synchronously. All the pellistors were tested in the above standard CH_4_ gas with different concentrations and tested in 50% LEL CH_4_ gas for 6 h every day. 

After aging treatment of 150 h in the environment of 50% LEL CH_4_, the pellistor was connected in the direct current bridge with temperature compensation, as is shown in Figure 9b. The temperature compensation element (*R_c_*) had the same diameter of matrix and catalytic volume as the sensing element. The operating temperature was 450 °C. The steady signal of the pellistor with an original signal of 140 mV was tested and remained at 80 mV. The output signal Δ*U* can be calculated by Equation (4):

(4) $$\Delta U=\frac{R_{s}-R_{c}}{2\left(R_{s}+R_{c}\right)}E$$

**Figure 11:**
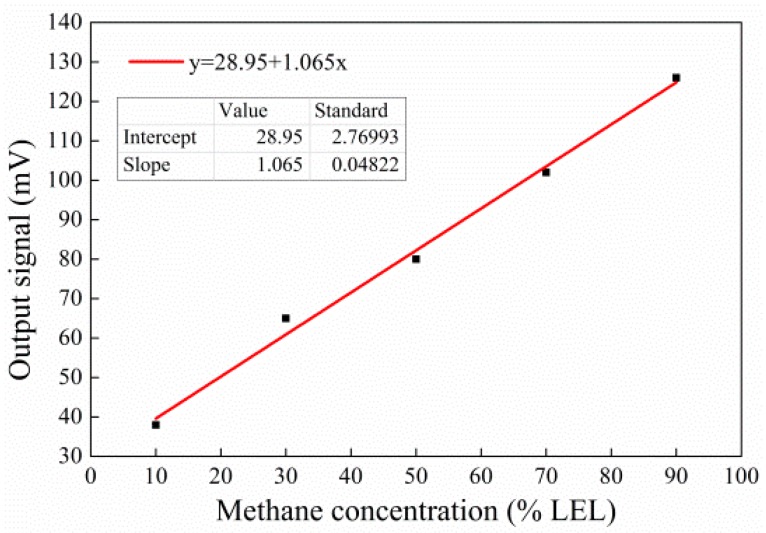
Variation in output signal of the sensor with variable methane concentrations.

The output voltage value for per unit gas concentration was used to represent the sensitivity of the sensor. The unit of sensitivity was mV/% CH_4_ for methane gas detection. The variation in output signal of the sensor versus variable methane concentrations is shown in Figure 11. All the data are the mean value of the above ten samples. While the methane concentration was in the range of 10% LEL CH_4_ to 90% LEL CH_4_, the output signal almost appeared to increase linearly. When the methane concentration was 50% LEL CH_4_, the output signal was 80 mV, according to Figure 11. The low explosion limit (LEL) in air for CH_4_ is 5%. This means that the output signal is 80 mV when CH_4_ concentration in air is 2.5%. Therefore, the sensitivity of the sensor could be calculated as 32 mV/% CH_4_. The larger the pellistor was, the greater the number of activated points in the catalytic material; the more activated points, the higher the sensitivity. In addition, the structure of the carrier after being sintered was filled without any defects in the shrinkage cavity via droplet generating technology. The sensitivity of 32 mV was relatively higher than that presented by Liu et al. [20]. 

#### 3.2.4. Power Consumption

Power consumption is another important performance index, because thousands of sensors will be placed in a mine and all the sensors should work all the time for several years. When pellistor diameter and catalyst volume were 450 μm and 16.0 nL, respectively, the original signal obtained the maximum value of about 140 mV, which means that this pellistor had the best output performance among all the tested samples. After aging treatment, the output signal remained 80 mV, and the power consumption was 120 mW. The power consumption of pellistors only demonstrating the best output performances were measured. This can be calculated by Equation (5):

(5) $$P=I^{2}\times R_{s}$$

where *I* is the current applied to the sensing element, and *R_s_* is the resistance value of the sensing element under working conditions. Experimental results have shown that the power consumption for the above sensor with the output signal of 80 mV was about 120 mW. The power consumption of the sensor was relatively high. The structure of the carrier after being sintered was filled without any defects in the shrinkage cavity via droplet generating technology. More energy was needed to heat the sensing element to the working temperature because the sensor in this paper had a slightly larger volume.

## 4. Conclusions

This paper presents a new method which uses a droplet generator based on pulse inertia force to introduce carrier and catalytic materials into a platinum coil and fabricate a micropellistor. The fabrication process of the pellistor includes four steps, which are adding Al_2_O_3_ suspension, forming the carrier, adding catalytic material, and forming the pellistor. The added amounts of both the carrier and catalytic materials can be controlled by the volume and rate of the ejected droplets. A bridge circuit is used to get the output signal of the gas sensors. Variation in the original signal of the pellistor with porous matrix size and amount of Pd-Pt catalyst was researched. The maximum value of original signal is above 140 mV when the pellistor diameter and catalyst volume are 450 μm and 16.0 nL, respectively. The steady output signal after aging treatment almost appeared to increase linearly with the increase of the methane concentration. The sensitivity and power consumption of the pellistor are 32 mV/% CH_4_ and 120 mW, respectively.
